# Imiquimod cream efficacy in the treatment of periocular nodular basal cell carcinoma: a non-randomized trial

**DOI:** 10.1186/s12886-015-0024-0

**Published:** 2015-04-03

**Authors:** Erick Marcet Santiago de Macedo, Rachel Camargo Carneiro, Patricia Picciarelli de Lima, Breno Gonçalves Silva, Suzana Matayoshi

**Affiliations:** Departments of Ophthalmology and Pathology, University of São Paulo School of Medicine, Av. Dr. Arnaldo, 455 São Paulo, Brazil; Departments of Pathology, University of São Paulo School of Medicine, Av. Dr. Arnaldo, 455 São Paulo, Brazil

**Keywords:** Basal cell carcinoma, Periocular neoplasm/therapy, Antineoplastic drug, Immunotherapy, Topical administration

## Abstract

**Background:**

The recurrence rate of periocular nodular basal cell carcinoma (PNBCC) following treatment with imiquimod (IMQ) has not yet been established. Previous studies did not include histological follow-up. The aim of this analysis was to evaluate the efficacy of topical immunotherapy with 5% IMQ cream for the treatment of PNBCC.

**Methods:**

Study design: A prospective, non-randomized, and uncontrolled longitudinal case series study. No participants were blinded. Punch biopsy confirmed PNBCC patients were included at the Ophthalmology Clinic of São Paulo University Medicine School Hospital (from 2008 to 2012). Patients were treated with 5% IMQ cream once a day, 5 days per week, for 8–16 weeks. Standard lesion photographic documentation was done during the study. Three months after treatment ended, an image-guided biopsy was performed. Patients were followed at 6-month intervals and annually for control biopsies. Main outcome measures were clinical and histological clearance rates. Data were analysed by frequency distribution for qualitative group characteristics and central tendency measures for quantitative data.

**Results:**

Twenty-four patients met the inclusion criteria, 19 of whom remained until the end of treatment. The histological clearance rate was 89.5% and 84.2%, respectively, at 3 and 39.5 months. The 3-year histological clearance rate was 81.8% (9/11) for lesions >10 mm, and 100% (8/8) for lesions <10 mm. Three patients did not tolerate the side effects of the medication and left the study. Two patients were excluded for treatment interruption related to comorbidities.

**Conclusions:**

Our results indicated that 5% IMQ cream was a useful alternative treatment for NBBCC, especially for lesions <10 mm. IMQ also showed a significant neoadjuvant effect on lesions >10 mm.

**Trial registration:**

ClinicalTrial.gov Registration Dec 3, 2008: #NCT 00803907.

## Background

The incidence of basal cell carcinoma (BCC), the most common human neoplasm, has increased significantly worldwide over the past few years [1,2]. More than 80% of BCCs affected the neck and face, 20% of which were in the form of periocular tumours [3]. It was estimated that 30–40% of patients with BCC would develop one or more new lesions over the next 10 years [4].

BCC is the main cause of required reconstructive surgery in the periocular region. For facial BCC, Mohs surgery is considered the method with the best chance of cure, with 5-year recurrence rates of up to 6.5% [5]. Nodular BCC, the most common subtype in eyelids, has a surgical cure rate of 85–95% [6].

For cases in which surgery is not possible (multiple lesions, high surgical risk, and refusal of surgery), topical immunotherapy may be an alternative. Imiquimod (IMQ), an immune modulator, acts by stimulating innate and adaptive immunity and by inducing apoptosis in tumour cells [2]. The ideal mode of administration of IMQ remains uncertain. The drug has been applied once or twice per day or 3–7 times a week during 6–16 weeks with variable results [7,8].

IMQ use, as an alternative treatment for periocular BCC, was suggested using results from a small case series with an almost 100% reported cure rate [9-11]. However, no histological follow-up was provided in these studies. Leppala et al. [12] and Garcia-Martin et al. [13], however, demonstrated short-term (3 months) biopsy documentation after treatments. For periocular NBCC (PNBCC), Eigentler et al. showed residual tumours in one third of patients given IMQ three times per week for 8–12 weeks [8].

The purpose of the present study was therefore to evaluate clinically and histologically the effects of 5% topical IMQ cream on PNBCC by evaluating for residual tumour rate and recurrence.

## Methods

This was an interventional prospective, non-randomized, and uncontrolled longitudinal case series study, conducted between 2008 and 2012. The study followed the tenets of the Declaration of Helsinki, and was approved by the University of São Paulo Medical School Hospital Institutional Review Board Ethics Committee. All participants gave their informed consent. The ClinicalTrial.gov number was NCT00803907.

Patients with periocular biopsy diagnosed as NBCC were included in this study. We limited eyelid margin lesions up to 20 mm, and medial canthus lesions up to 30 mm (largest diameter), with no infiltrating deeper tissues on palpation. All lesions had not had prior treatment. Recurrent BCC lesions and patients with clinical signs of orbit invasion were excluded. Uncooperative patients with no caregiver assistance to correctly apply the medication were also excluded.

Main outcome measures were clinical and histological clearance rates. To detect a histological tumour clearance rate of 50% at 3 months, with a 5% significance level and 95% confidence, a sample size of 22 patients was necessary, given an anticipated dropout rate of 10% (http://www.nss.gov.au/nss/home.nsf/). All patients were submitted to IMQ cream treatment. No participants were blinded.

IMQ cream (Aldara®; Meda AB, Graceway Pharmaceuticals, Bristol, TN, USA) was applied once each day at bedtime. To ensure patient safety, we instructed patients and caregivers (or relatives) to use lubricating gel in the conjunctival sac before IMQ cream application. We stressed the correct medication application, using a swab, and taking care to keep the border of the eyelid away from the eye. The cream remained in contact with the tumour for 8 to 10 hours. In the morning, the periocular area was washed with neutral liquid soap. In cases of accidental contact with the ocular surface and conjunctiva, the patient was instructed to wash abundantly with saline solution and apply lubricating eye gel.

Artificial tears were prescribed and provided free of charge to the patients, for application every 6 hours during the day. IMQ was used once each day, 5 days a week, for a minimum of 8 weeks and a maximum of 16 weeks. Within this period, treatment was discontinued once the lesion became undetectable by slit lamp examination and palpation.

During the treatment period, patients were followed biweekly and information was collected through questionnaires, slit lamp examinations, visual acuity testing, photography, and measurement of lesions, using Image J software (version 1.42) [14].

Baseline measurements were obtained from photographs taken after the initial biopsy so that the amount of tissue removed would not influence the results. The final measurements were based on photographs taken 3 months (12 weeks) after treatment end.

We used 2-mm trephine for all biopsies. An image-guided biopsy of the region was performed 3 months (12 weeks) after treatment. Patients with clinical or histological findings of residual lesions were referred for surgical excision and reconstruction. Patients were followed at 6-month intervals, with annual control biopsies.

Data were analysed by frequency distribution for qualitative group characteristics and central tendency measures for quantitative data.

## Results

**Figure 1:**
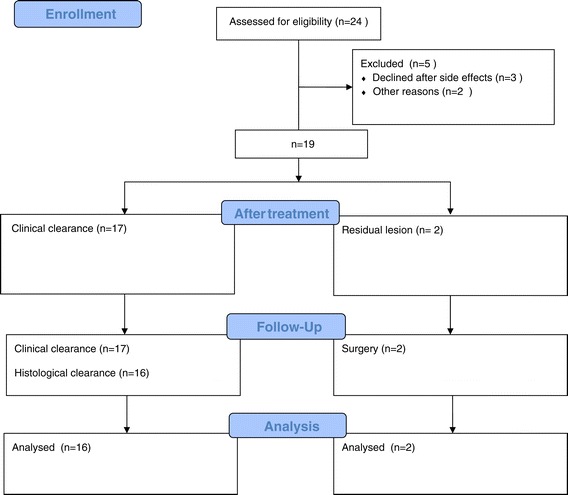
**Imiquimod (IMQ) treatment flow chart.**

Throughout the study, 24 patients met the inclusion criteria, 19 of whom remained until the end of treatment. Patients were recruited until December 2012 when the required sample size was obtained. Three patients did not tolerate the side effects of the medication and left the study. One patient suffered an ischemic cerebrovascular accident, and one patient died during the treatment period. Both cases were associated with previous diseases and high surgical risk (Figure 1).

**Figure 2:**
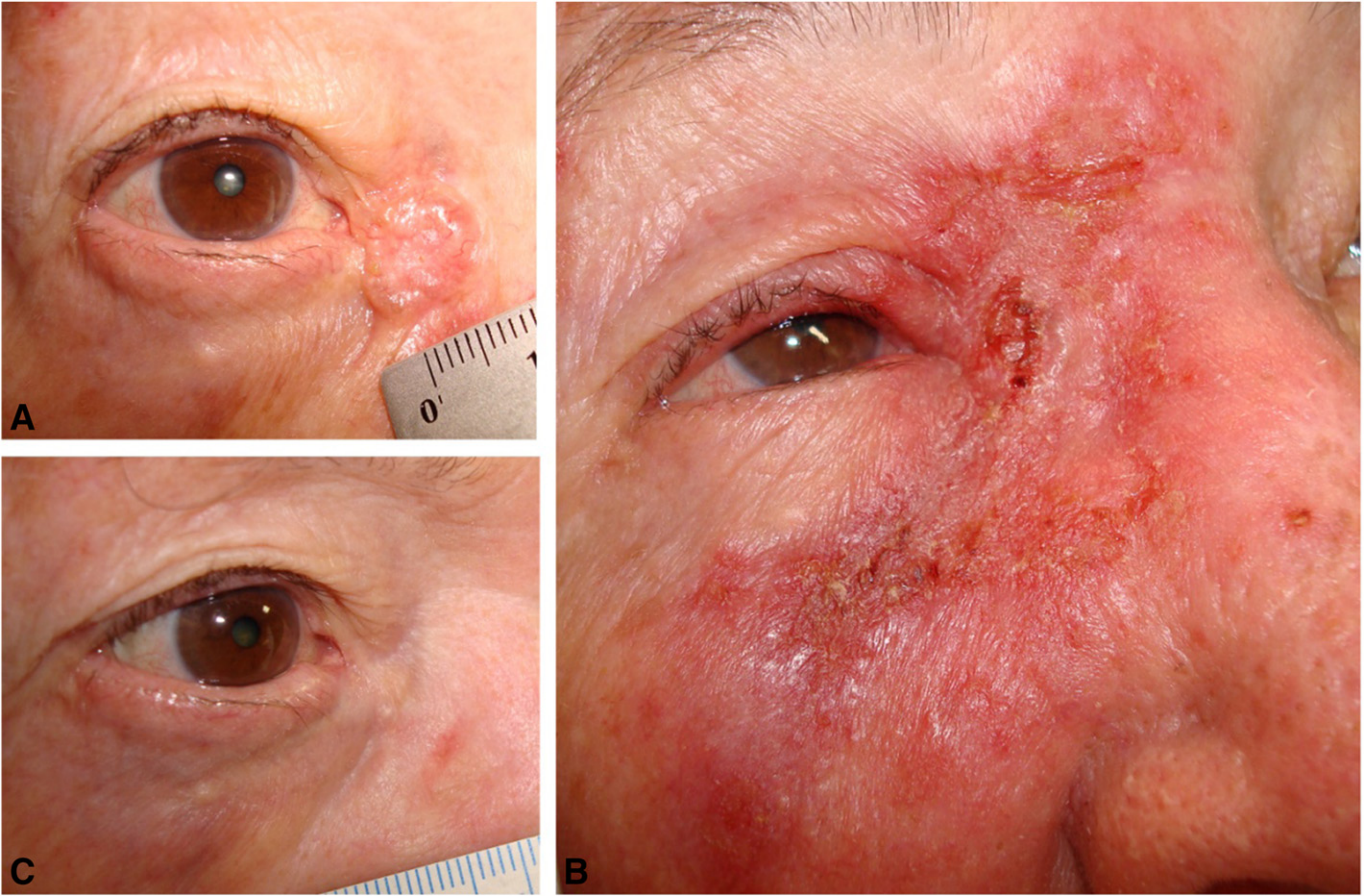
**Lesion #12. Nodular basal cell carcinoma in the medial canthus. A)** before treatment, **B)** local inflammation mimicking preseptal cellulitis, **C)** 2 years after treatment completion, when the biopsy identified BCC recurrence.

One patient (case 12) interrupted treatment for 2 weeks because of intense local inflammation associated with systemic symptoms. This patient was also the only patient who experienced tumour recurrence, as confirmed by biopsy 2 years after treatment (Figure 2).

**Figure 3:**
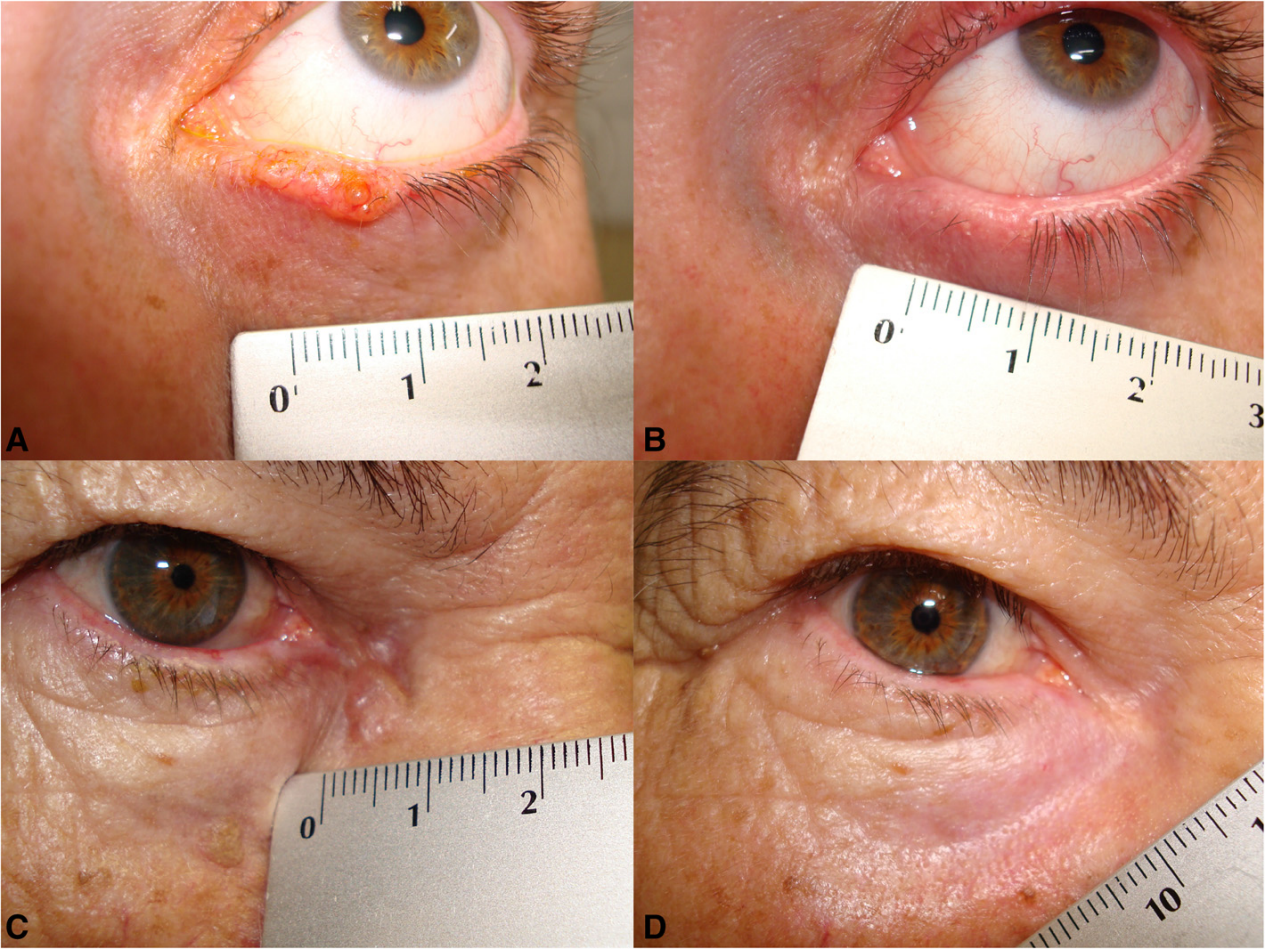
**Clinical pictures before and 3 years after treatment. A)** Case #13: Eyelid margin PNBCC, **B)** after treatment, **C)** case #4, medial canthus PNBCC, **D)** after treatment.

Seventeen patients had complete clinical clearance at the 3-month evaluation (Table 1). Two patients had a residual tumour (Table 2). Punch biopsy showed a histologically tumour-free rate of 89.5% at 3 months and 84.2% by the end of follow-up (39.5 months) (Table 1). Two cases with successful outcomes are shown in Figure 3.

**Table 1:** **Description of the lesions treated with 5% imiquimod cream**

**Variable**	**Frequency**	**%**
**Age (years)**		
<60	5	26.3
60–70	9	47.4
>70	5	26.3
**Gender**		
Male	13	68.4
Female	6	31.6
**Tumour site**		
Lower eyelid	8	42.1
Medial canthus	11	57.9
**Fitzpatrick scale**		
2	13	68.3
3	4	21.1
4	1	5.3
5	1	5.3
**Clinical cure at 3 months**		
Yes	17	89.5
No	2	10.5
**Histological cure at 3 years**		
Yes	16	84.2
No	3	15.8
**Total**	19	100

**Table 2:** **Description of 19 patients with confirmed periocular basal cell carcinoma treated with 5% imiquimod cream**

**Lesion**	^**†**^ **Area (mm** ^**2**^ **)**	^**†**^ **Largest diameter (mm)**	^**‡**^ **Residual area (mm** ^**2**^ **)**	^**‡**^ **Largest residual diameter (mm)**	**Treatment time (weeks)**
**1**	131.97	12.01	13.53	4.25	8
**2**	108.18	11.70	0	0	12
**3**	64.56	10.63	0	0	16
**4**	67.09	11.65	0	0	12
**5**	53.34	15.03	0	0	12
**6**	20.76	5.10	0	0	8
**7**	32.32	7.51	0	0	10
**8**	165.38	27.18	22.07	7.63	12
**9**	189.14	17.96	0	0	12
**10**	18.53	5.06	0	0	14
**11**	15.99	2.77	0	0	12
**12**	121.01	15.66	0	0	16
**13**	35.53	9.46	0	0	14
**14**	45.32	16.01	0	0	12
**15**	4.36	2.82	0	0	12
**16**	39.83	10.29	0	0	14
**17**	121.27	15.45	0	0	16
**18**	60.63	8.70	0	0	16
**19**	32.18	5.38	0	0	12

^†^Before treatment, ^‡^3 months after treatment completion.

The most frequent ocular symptoms and signs were conjunctivitis (95%), followed by keratitis (84%), foreign body sensation (79%), lacrimation (58%), low visual acuity (53%), and ectropion (37%). Treatment consisted of frequent lubrication with eye drops. All patients presented with some degree of skin reaction such as hyperaemia, crusting, ulceration, and bleeding during treatment. No medication was prescribed except for cold compresses. No side effects were permanent and all were resolved after the end of treatment.

## Discussion

The current investigation confirmed several previous studies reporting favourable results with IMQ [12,13] and suggested that IMQ played an important role in the management of PNBCC as an alternative non-surgical treatment. The present study proposed treatment once per day, five times per week for 8 to 16 weeks. Treatment time depended on healing of skin and absence of tumours at slit lamp examination, and on palpation, therefore treatment lasted 12.6 weeks on average. Histological clearance was 89.5% (17/19) by the end of the treatment, and 84.2% (16/19) after 3 years of follow-up (Table 2).

Previous PNBCC studies reported treatments lasting 6 weeks, at seven applications per week and with 71% efficacy, and 50–59% efficacy with three applications [15,16]. However, three applications per week for 8 weeks yielded an efficacy of 64% [8]. The highest cure rates reported were observed with treatment for 12 weeks as follows: 60–63% efficacy with three applications per week, 70% efficacy with five applications per week, and 76% efficacy with seven applications per week [8,16].

In the present study, 57.9% (11/19) of the patients had lesions >10 mm. Interestingly, the 3-year histological clearance rate was 81.8% (9/11) for lesions >10 mm, and 100% (8/8) for lesions <10 mm. Eigentler et al. obtained the best results in smaller lesions (72% cure rate for smaller lesions versus 48% for larger lesions), and observed that the larger the tumour, the less efficacious the medication and the longer the required treatment [8].

Thus, a treatment duration of 6 weeks was reported to be a negative prognostic factor in the treatment of periocular lesions larger than 10 mm [13]. This finding was supported by the association between tumour profile (percentage of tumours greater than 10 mm and mean and median lesion size) and different dosage regimens used in six studies evaluating treatment of PNBCC with 5% IMQ cream (Table 3) [9-13,17]. Partial resolution (tumours were reduced but did not disappear completely, producing a neoadjuvant effect) was more commonly observed with nodular lesions when compared with superficial BCC [18].

**Table 3:** **Comparison of results evaluating imiquimod treatment of periocular nodular basal cell carcinoma**

**First author year**	**N**	**Dosing regimen, days/week, weeks**	**Mean size (SD) mm**	**Lesions greater than 10 mm (%)**	**Complete response (%)**	**Follow-up (years)**	**Histological follow-up (months)**
**Present study**	19	5/wk	11.1 (6.0)	58	84.2	3.3	36
		8–16 wk					
**Prokosch 2011** [11]	4	5/wk	11.5 (4.5)	50	100	7.0	-
		6 wk					
**Garcia-Martin 2010** [13]	15	5/wk	7.6 (2.9)	25	100	2.0	3
		6 wk					
**Carneiro 2010** [22]	10	5/wk	12.1 (6.5)	60	80	1.0	12
		10–16 wk					
**Choontanom 2007** [10]	5	5/wk	10.8 (4.2)	40	80	3.0	-
		6 wk					
**Leppälä 2007** [12]	4	5/wk	8.5 (4.6)	25	100	0.5	3
		6 wk					
**Blasi 2005** [9]	2	3/wk	-	-	100	1.0	-
		8–12 wk					

SD = standard deviation; wk = weeks.

Only two of our patients had partial tumour clearance. Both had lesions larger than 10 mm, and in both cases the medication had a neoadjuvant effect, significantly reducing tumour size and thereby facilitating surgery (Table 1). The less healthy the tissue removed in surgery, the smaller the risk of compromising eyelid function and aesthetics. The main complications of surgical excision of large amounts of periocular tissue included scarring, palpebral retraction, trichiasis, ptosis, chronic epiphora, entropion, ectropion, keratitis, and corneal ulcer caused by exposure or perforation [19].

At baseline, lesion 1 (lower eyelid margin) measured 13.5 mm. Adding a 2-mm safety margin on each side, an area equivalent to half the eyelid (17.5 mm), would have required reconstruction of the eyelid. After treatment with 5% IMQ cream, the diameter was reduced to 4.2 mm. Including the safety margin, the area to be reconstructed (8.2 mm) was less than one third the size of the eyelid. In this patient the safety margins were free and the lesion was submitted to pentagonal excision, cantholysis, and closure.

Lesion 8 (medial canthus) was reduced from 27.2 mm to 7.6 mm. Thus, instead of performing a complex surgery with glabellar flap rotation and advancement, the patient was submitted to simple excision and direct closure of the tumour-free surgical margins.

The patient with lesion 12 interrupted the treatment for 2 weeks because of intense local inflammation mimicking preseptal cellulitis, but systemic symptoms were also observed, especially diarrhoea. The interruption may have influenced the evolution because this was the only patient with recurrence of BCC (Figure 1). There was evidence that recurrence of superficial BCC was low after treatment with IMQ and that the resolution rate remained high during 5 years of clinical follow-up. The clinical and histological outcome 12 weeks after treatment appeared to be a reliable predictor of the risk of recurrence, which was most commonly observed during the first year of follow-up [20].

Bath-Hextall et al. recently reported a large randomized trial with 3 years of follow-up comparing IMQ and surgery in nodular and superficial BCC, showing a recurrence rate of 18.3%. However, this study did not include periocular regions [21]. The recurrence rate of PNBCC following treatment with IMQ has not yet been established. Studies with a larger series of BCC patients have focused on short-term elimination of tumours rather than on the observation of recurrence. We identified only one study with a long enough follow-up time to detect recurrence, a study of four patients with BCC in the periocular area, followed up for 7 years [11]. Two studies performed biopsy at 3 months [12,13]. Because PNBCC is more complex and more established than superficial BCC, late recurrence without clinical signs is a possibility, and was observed for lesion 12 (Figure 1–C), which recurred after 2 years.

Our study was the largest IMQ-treated PNBCC patient series with histological follow-up beyond 3 years. Follow-up for recurrence was based on clinical examinations and histological controls. Excisional biopsy of whole compromised areas would be desirable but not feasible to perform every year. The 2-mm punch biopsy used here was a swift and simple diagnostic technique with a high level of agreement compared with analysis of whole surgical specimens (90% accuracy for malignancy diagnosis) [22].

Despite longer treatment, our patients had only minor ocular symptoms (conjunctivitis, keratitis, and foreign body sensation) and no permanent eye damage was observed. Cannon et al., in a retrospective study, described conjunctivitis and eye burning sensation as the most common symptoms during treatment of periocular lesions, despite the fact that only three in a sample of 47 patients had periocular BCC, and IMQ was administered three times per week for 4–6 weeks [23]. All symptoms caused by the medication were transient and resolved after ending treatment.

The main limitation of the present study was the lack of a comparative group (such as a surgical group). Regarding generalization to other cases, our cases consisted of primary PNBCC lesions without signs of deep infiltration or orbit invasion, therefore it was a limited group of patients.

Although surgical excision remains the gold standard for PNBCC and is associated with the highest cure rates [24], in the present study IMQ was shown to be an efficacious and safe alternative treatment. However, we stress the importance of careful follow-up even after clinical clearance to manage recurrence cases.

## Conclusions

The recurrence rate of periocular nodular basal cell carcinoma (BCC) following treatment with IMQ was low. The 3-year histological clearance rate was 100% (8/8) for lesions <10 mm and 81.8% (9/11) for lesions >10 mm. Importantly, IMQ had significant neoadjuvant effects on PNBCC lesions >10 mm.
